# Apert syndrome: a rare clinical image

**DOI:** 10.11604/pamj.2023.45.24.38946

**Published:** 2023-05-08

**Authors:** Chundi Sai Samhitha, Chiruvella Subramanyam

**Affiliations:** 1 Department of Pediatrics, Jawaharlal Nehru Medical College, Datta Meghe Institute of Higher Education and Research, Wardha, Maharashtra, India,; 2 Department of Paediatrics, Christian Medical College, Dr. M.G.R Medical University, Chennai, Tamil Nadu, India

**Keywords:** Syndactyly, fingers and toes, Apert syndrome

## Image in medicine

Apert syndrome is a rare congenital genetic deformity in which early fusion of skull bones, face, and limbs, hence this condition is also known as acrocephalosyndactyly. Patients with Apert syndrome typically have craniosynostosis, midface hypoplasia, and syndactyly. A 6-year-old female child was brought to our hospital with a fever for 1 day. On examination, the patient had hypertelorism, a steep forehead, a small nose, a depressed nasal bridge, flattened occiput (A), syndactyly of both hands with complete fusion of second, third, fourth, and fifth fingers (B) and syndactyly of toes (C) were seen. The fused fingers and toes had separate nails. There is no cleft lip or palate in this patient. Other symptoms like the cardiovascular system are normal and no murmur is heard. There were no abnormal findings in the echocardiogram and ultrasonography. Treatment includes surgery to release syndactyly fingers for improving functionality. Speech therapy should be started early for a better outcome.

**Figure 1:**
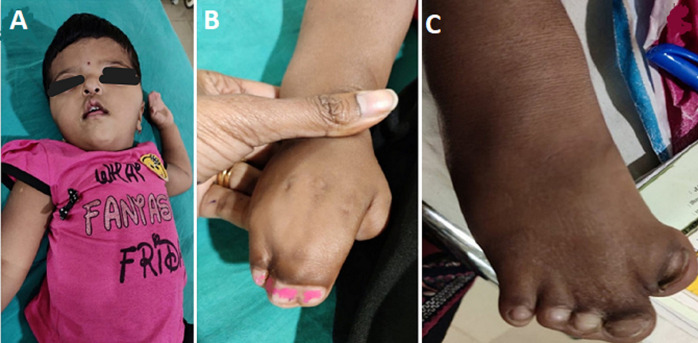
A) Apert syndrome facial deformity; B) syndactyly of fingers; C) syndactyly of toes

